# Meetings of Societies

**Published:** 1912-09

**Authors:** 


### MEETINGS OF SOCIETIES.

### Bristol Medico-Chirurgical Society.

*April 10th*, 1912.

Mr. C. A. Morton, President, in the Chair.

Dr. J. Odery Symes opened a discussion on **The diagnosis of the causes of chronic recurrent abdominal pain.** The causes of chronic and recurrent abdominal pain are so numerous that time will only permit to mention many of them, passing on then to the consideration of those presenting the greater difficulties. The headings of his address are as follows:—

(1) *Stomach, duodenum, and intestinal appendix.*
Functional. Obstructive.
Inflammatory. Malposition.
Ulcerative.

(2) *Pancreas and liver and gall-bladder.*
Inflammatory.
Calculi.
Tumour.

(3) *Renal.*

Aneurysm of aorta or mesenteric arteries, diseases of spinal column, cord and membranes (tabes, caries, psoas abscess), lead colic, hysteria, neurasthenia, and diseases of the pelvic organs.

Dr. WALTER SWAYNE dealt with the incidence and symptomatic value of abdominal pain in **diseases of the female generative organs.** He called attention to the localisation of the different pathological conditions by the pain to which they gave rise, and to the way in which inflammation of the appendix on the right side was apt to be associated with inflammatory affections of the right appendages, while on the left diseases of the sigmoid might lead to mistaken diagnosis of disease of the left ovary and tube. He alluded to the fact that uterine displacements, especially retroversion of the uterus, might give rise to pain in the hypogastrium. He also pointed out how in ovarian tumours and fibroid tumours of the uterus abdominal pain was an important indication of such accidents as twisted pedicles in the one and degeneration in the other. The occurrence of abdominal pain in pelvic inflammation was also alluded to, and its special characteristics described. The characteristics of the abdominal pain due to ectopic gestation were also noticed, and attention drawn to its sudden onset and engravement character. The characters of the pain produced in pregnancy by what is probably rheumatic myositis were also dealt with, and it was shown that acute abdominal pain during pregnancy, constant in type and without febrile symptoms, was extremely rare, except as the concomitant of some gross pathological lesion, such as tumour of the gravid uterus of or the pedicle of an ovarian tumour existing with pregnancy, or of concealed accidental hemorrhage. The pain due to carcinoma of the body of the uterus was also alluded to, as well as to the acute pain produced by a stone in the ureter, which might be mistaken for oöphoritis.

Dr. RUPERT WATERHOUSE referred to the condition known as appendix dyspepsia, of which he had seen two cases in young girls. There was pain and vomiting after food, but laparotomy revealed appendical adhesions and concretions to be the probable cause. He thought surgeons were apt to overlook the gastric crises of tabes and lead poisoning as causes of abdominal pain. He had, however, seen cases in which gastric ulcer had followed lead colic, and in one case of the kind, with perforated gastric ulcer, an operation was not performed, because the patient was known to have suffered from lead colic. The speaker had no belief in the "moral effect" of operation in hysteria, and considered operation should be avoided in abdominal hysteria. He thought the signs and symptoms of that form of abdominal trouble were not sufficiently taught. The patients were frequently alert, vivacious women, and complained of stomach pain and vomiting. There was no rigidity and no hyperalgesia. Deep pressure into subjacent structures elicited pain, which seemed to lie along the aorta and iliac vessels.—Mr. CARWARDINE referred to the surgical causes of abdominal pain, and gave the following classification: (1) Colics of internal origin, (2) mesenteric and omental twists, (3) inflammation of organs, when the inflammation spread to the peritoneum, (4) impaction of viscera, (5) thrombosis and embolism of vessels. He pointed out that the organs themselves are generally painless, but that the mesenteric attachments and parietal and visceral peritoneum are susceptible to pain. Referred pains cannot be associated with any particular organ unless at some time or other that organ is tender. Conversely, for accurate diagnosis, the local tenderness should correspond with definite referred pains. The application of the foregoing to specific instances was then detailed. Gastric ulcer may be present without pain. The so-called "hunger pain" is associated with lesions in the right hypochondrium involving the peritoneum, not alone with duodenal ulcer. The pain of gall-stones, renal mobility and calculus, appendicitis, intestinal colic, twisted ovarian pedicles, etc., was discussed on the same lines.—Dr. MICHELL CLARKE said that if severe abdominal pain and tenderness were present the cause was something more than dyspepsia. In obscure cases of pain a "lead line" should be looked for and the knee-jerks tested. A patient with chronic recurrent abdominal pain gradually becomes neurotic. Mucous colitis as a cause of pain had to be remembered, also abdominal pain due to chest conditions, *e.g.* unresolved pneumonia.—Dr. JAMES SWAIN thought the subject too large to admit of a full discussion of the many causes of chronic and recurrent abdominal pain, which had been so well classified by Dr. Symes. He agreed that the most common causes of such pain were usually found in association with the appendix, kidney and gall-bladder. These, however, rarely presented any difficulty. The tenderness at McBurney's point in the case of the appendix, the reflection of the pain along the last dorsal, ilio-inguinal and ilio-hypogastric nerves in affections of the kidney, and the sub-costal tenderness in the case of the gall-bladder, were very helpful in diagnosis in these several conditions. Many cases of chronic and recurrent pain were due to some form of intestinal adhesions, and here a history of former peritonitis—tuberculous or simple—of hernia, operation, or appendicitis would aid us in coming to a conclusion as regards the probable origin of the trouble. Mucous colitis must also be thought of, and he (the speaker) had occasionally been consulted in such cases with a view to operation for supposed disease of the appendix. An examination of the stools would prevent error. There was one class of case to which reference had not been made, namely simple colic, which is often very difficult to differentiate from intestinal adhesions or malignant growth. He had seen many cases where the recurrent pain, constipation, abdominal distension, and even wasting and occasional vomiting, which may occur in cases of colic, caused great difficulty in arriving at an accurate diagnosis. The occurrence of occult blood in the fæces would be suggestive of an ulcer either simple or malignant, but he was not inclined to give much weight to the fact of retardation of a bismuth meal as an aid to the diagnosis of obstruction from malignant growth. Many cases of simple colic could only be diagnosed from malignant disease by carefully watching the patient in bed. If in spite of the relief of constipation and careful dieting, the patient steadily lost weight the abdomen should be explored, when an early malignant neoplasm would probably be found; but if improvement took place under these conditions, the case would probably be one of simple colic, and we could justifiably avoid surgical interference.—Mr. BRASHER agreed that a thorough exploration in obscure cases should be made by laparotomy, but opposed the advice to use a transverse incision.

---

